# A tale of Rabs and the exocyst complex in ciliary trafficking and biogenesis

**DOI:** 10.3389/fcell.2025.1574638

**Published:** 2025-05-06

**Authors:** Priyanka Upadhyai, Debalina Bose, Neha Quadri

**Affiliations:** ^1^ Department of Medical Genetics, Kasturba Medical College, Manipal, Manipal Academy of Higher Education, Manipal, Karnataka, India; ^2^ Advanced Technology Development Centre, Indian Institute of Technology Kharagpur, Kharagpur, West Bengal, India

**Keywords:** primary cilia, Rabs, Rab-like proteins, exocyst, ciliary trafficking, ciliopathies

## Abstract

The primary cilium is a microtubule-based sensory cell organelle templated by a modified parent centriole that mediates mechanotransduction and response to biochemical cues such as morphogens to regulate organismal development and homeostasis. Given that the cilium is a specialized microdomain devoid of its translation machinery, it relies on the endomembrane pathway for the delivery of proteins and other biomolecules to it. This review provides a comprehensive insight into how membrane trafficking modulators such as Rab and Rab-like proteins, and the exocyst complex control transport to the primary cilia, in turn regulating various aspects of their assembly and function. We integrate findings from *in vitro* and animal models and draw on human diseases associated with the dysfunction of Rabs or exocyst that exhibit phenotypes overlapping with those of ciliopathies, which further support their relevance to cilia biogenesis and maintenance.

## 1 Introduction

Cilia are conserved microtubular cell organelles indispensable for organismal development and tissue homeostasis. They were present in the last eukaryotic common ancestor (LECA) and have evolved a variety of structural specializations congruent with their functional adaptations throughout metazoa (Carvalho-Santos et al., 2011; Cavalier-Smith, 2022; Leung et al., 2025). Despite their architectural diversity, they retain universal features, such as an axonemal backbone with nine-fold microtubular symmetry that is templated by the mature parent centriole or basal body and is encased within the ciliary membrane that is contiguous with the plasma membrane. Cilia can be categorized into two broad groups: motile and primary cilia.

Motile cilia contain an axoneme with a central pair of microtubules in addition to the nine outer microtubule doublets (9 + 2) and are involved in cell functions such as fluid propulsion or cell motility. In more complex metazoans, they are present in the brain, middle ear, lungs, male and female reproductive tracts, and sperm, modulating functions such as the flow of cerebrospinal fluid, hearing, left-right patterning, mucus clearance, and mobility of the ova and sperm. Primary cilia are present in many postmitotic eukaryotic cells. They are non-motile due to the absence of the central pair of microtubules in their axonemes (9 + 0), except those at the left-right organizer in vertebrate embryos (Amack, 2022). Defects in ciliary structure or function cause disorders termed ciliopathies, which often manifest as congenital multisystemic abnormalities with phenotypes such as kidney cysts, retinal degeneration, and skeletal defects (Reiter and Leroux, 2017).

Cilia are largely devoid of translation machinery, except for mouse ependymal cells (Hao et al., 2021). During their assembly, membrane expansion is coordinated with axonemal extension; consequently, they rely on importing building blocks, such as tubulin (Craft et al., 2015), with the help of the intraflagellar transport (IFT) machinery (Lacey and Pigino, 2024). Proteins destined for the cilium contain ciliary targeting sequences (CTSs) and are transported to the cilium or periciliary membrane at its base by polarized vesicle trafficking (Nachury et al., 2010; Hsiao et al., 2012). This involves the biogenesis, trafficking, tethering, and fusion of vesicles that are orchestrated by a battery of factors, such as coat proteins, Ras-related protein in brain (Rab) guanosine triphosphatases (GTPases), multisubunit tethering complexes (MTCs), soluble N-ethyl-maleimide-sensitive factor (NSF) attachment protein receptors (SNAREs), motor proteins, and adaptors. The delivery of proteins into the ciliary compartment involves CTS recognition by specific mechanisms. Apart from some CTSs, for example, the Ax(S/A)xQ sequence recognized by the BBSome during the ciliary trafficking of somatostatin receptor 3 (Sstr3) (Jin et al., 2010), CTSs and corresponding trafficking module for most cilia proteins remain to be elucidated.

While membrane and soluble proteins (<40 kDa) can be passively translocated across the ciliary diffusion barrier, their delivery into the ciliary compartment is selectively modulated by the IFT-BBSome machinery, for example IFT-B regulates the transport of radial spokes in *Chlamydomonas* flagella (Lechtreck et al., 2022). Alternatively, importins can function as ciliary trafficking receptors for example, importin-β1 serves as the receptor for the transport of a Crumbs3 isoform to the primary cilium (Fan et al., 2007). Mechanisms of ciliary protein targeting have been extensively reviewed elsewhere (Jensen and Leroux, 2017; Lu and Madugula, 2018).

Here we review the Rabs, Rab-like proteins, and the exocyst complex, which is an MTC involved in vesicle tethering and exocytosis primarily at the plasma membrane, on how they regulate trafficking to the cilium, thereby controlling its biogenesis, maintenance, and function. Mutations in genes encoding these proteins or their regulatory factors may lead to disorders with abnormal cilia, underscoring their significance in cilia assembly and related pathologies (Table 1).

**TABLE 1:** Genetic disorders linked to cilia-associated Rabs and the exocyst complex.

Gene	Disease	Inheritance	OMIM #	References
Rabs				
*RAB23*	Carpenter syndrome; CRPT1	AR	201000	Taravath and Tonsgard (1993), Kadakia et al. (2014)
*RAB28*	Cone-rod dystrophy 18; CORD18	AR	615374	Roosing et al. (2013), Riveiro-Álvarez et al. (2015)
*RAB34*	Orofaciodigital syndrome XX; OFD20	AR	620718	Bruel et al. (2023), Batkovskyte et al. (2024)
*RAB35*	Developmental delay, hydrocephalus, Dandy-walker malformation, axial hypotonia, peripheral hypertonia, vision and hearing deficiencies	AR	-	Aguila et al. (2024)
*IFT27/RABL4*	Bardet-Biedl syndrome 19; BBS19	AR	615996	Aldahmesh et al. (2014)
	Lethal Fetal Ciliopathy	AR	-	Quélin et al. (2018), Haïm et al. (2025)
Exocyst complex				
*EXOC2*	Neurodevelopmental disorder with dysmorphic facies and cerebellar hypoplasia; NEDFACH	AR	619306	Van Bergen et al. (2020)
*EXOC4*	Nephrotic syndrome	AD	-	Nihalani et al. (2019)
*EXOC6B*	Spondyloepimetaphyseal dysplasia with joint laxity, type 3; SEMDJL3	AR	618395	Girisha et al. (2016), Campos-Xavier et al. (2018), Simsek-Kiper et al. (2022)
*EXOC7*	Neurodevelopmental disorder with seizures and brain atrophy; NEDSEBA	AR	619072	Coulter et al. (2020)
*EXOC8*	Neurodevelopmental disorder with microcephaly, seizures, and brain atrophy; NEDMISB	AR	619076	

AD: autosomal dominant; AR: autosomal recessive.

## 2 Cilia structure and compositional uniqueness

The ciliary compartment is a specialized domain, enriched with biomolecules required for its assembly and functions. It is subdivided into distinct functional subdomains: ciliary pocket, a periciliary membrane invagination separating the membranes of some cilia from the adjoining plasma membrane (Molla-Herman et al., 2010); basal body anchoring it to the cell body through distal appendages (DAs) (Tanos et al., 2013; Ye et al., 2014) and subdistal appendages (sDAs) (Mazo et al., 2016; Chong et al., 2020); transition zone (TZ), the axonemal region most proximal to the basal body containing Y-shaped linkers that connect it to the ciliary membrane at sites known as ciliary necklace (Park and Leroux, 2022). The TZ consists of at least 15 proteins and is organized into the Meckle Syndrome (MKS), Nephronophthisis (NPHP), and Core-scaffolding modules (Park and Leroux, 2022). In metazoans, the subdomain proximal to it is termed the Inversin (INV) compartment; it has a distinct composition; it is devoid of Y-links and interacts with the TZ physically (Otto et al., 2003) and functionally (Warburton-Pitt et al., 2012). The distal appendage proteins (DAPs) form a cone-shaped gate in the transition fibers (TFs) that are modified DAs at the cilium base (Reiter et al., 2012), wherein CEP83, CEP89, CEP164, and SCLT1 form pinwheel spokes and FBF1 is embedded in the matrix (Yang et al., 2018). The ciliary tip is the distalmost end of the cilium where components of signaling pathways, such as, Glioma (Gli) and Sufu from the Hedgehog (Hh) pathway, aggregate in a signal-dependent manner (Haycraft et al., 2005; Chen et al., 2011) and retrograde cargo is loaded (Ye et al., 2018b). When G-protein-coupled-receptors (GPCRs) fail to be retrieved back to the cell via the BBSome (Ye et al., 2018a), they accumulate at the cilia tip before being removed by extracellular vesicles (Nager et al., 2017; Phua et al., 2017).

The cilium houses receptors and components of many signaling pathways, including Hh, GPCR, receptor tyrosine kinase, calcium, and transforming growth factor pathways, that enable it to integrate and transduce a plethora of extracellular signals, including developmental morphogens and mechanical cues (Mill et al., 2023; Hilgendorf et al., 2024). The spatiotemporal modulation of ciliary composition is integral to its dynamic signaling output and is regulated by the gating properties of the TZ and TFs (Chih et al., 2011; Jensen et al., 2015; Yang et al., 2018). In addition, protein distribution in the ciliary membrane (Hu et al., 2010) and along the axoneme (Ghossoub et al., 2013) is modulated by macromolecular scaffolds formed by the Septin proteins.

Investigation of primary cilia from various types of human and rodent cells (Ostrowski et al., 2002; Liu et al., 2007; Mayer et al., 2009; Ishikawa et al., 2012; Narita et al., 2012), motile cilia from multiciliated vertebrates (Sim et al., 2020) and unicellular species (Smith et al., 2005; Subota et al., 2014; McCafferty et al., 2024) shed light on their unique proteomes. These studies demonstrate that different ciliary subdomains also vary in their membrane lipid composition, which in turn is distinct from that of the plasma membrane. While the ciliary membrane has high levels of phosphatidylinositol-4 phosphate (PI4P) (Chávez et al., 2015; Garcia-Gonzalo et al., 2015), the TZ (Conduit et al., 2024) and plasma membrane (Kanemaru et al., 2022) are enriched with phosphatidylinositol 3, 4, 5 triphosphate (PI(3,4,5)P_3_) and phosphatidylinositol 4,5 bisphosphate (PI(4,5)P_2_), respectively. This unique lipid profile of the ciliary membrane subdomains is achieved by the opposing actions of phosphatases, for example, inositol polyphosphate 5 phosphatase E (INPP5E) (Bielas et al., 2009; Jacoby et al., 2009) and kinases, for example, type Iγ phosphatidylinositol 4-phosphate 5-kinase (PIPKIγ) (Xu et al., 2016). INPP5E localizes at the axoneme and its activity leads to the hydrolysis of 5-phosphate in PI(4,5)P_2_, leading to PI4P enrichment in the ciliary membrane; PI(4,5)P_2_ accumulates at the TZ owing to PIPKIγ localization at the basal body (Conduit and Vanhaesebroeck, 2020). The selective compartmentalization of phosphoinositides plays a pivotal role in modulating ciliary signaling. Tulp3, a PI(4,5)P_2_-interacting protein binds IFT-A complex to promote trafficking of a subset of ciliary GPCRs, such as Sstr3, Mchr1 (Mukhopadhyay et al., 2010) and Gpr161 (Chávez et al., 2015; Garcia-Gonzalo et al., 2015). Subsequently, Tulp3 has been shown to regulate a generalized multistep process for the ciliary uptake for integral membrane proteins (Badgandi et al., 2017). The spatial organization of phosphoinositides in the cilium also helps in regulating its morphology, for example, ribosome profiling of cilia regeneration in *Chlamydomonas* revealed serine palmitoyltransferase as an essential modulator of cilia morphology and biogenesis, as ceramides produced by it bind with IFT particles and motor proteins to mediate axoneme and ciliary membrane interaction (Wu et al., 2022).

## 3 Rabs in cilia trafficking

Rab proteins belong to the Ras superfamily of small GTPases, with 11 and 70 members identified in budding yeast and humans, respectively. They regulate various steps of intracellular trafficking, such as vesicle budding, transport, tethering, and membrane fusion. Functionally, they cycle between active GTP-bound and inactive GDP-bound forms (Müller and Goody, 2018). In their active state, they are engaged at the membrane via C-terminal prenylation and interact with specific effector proteins. The guanine nucleotide exchange factors (GEFs), promote the exchange of GDP for GTP, conversely, GTPase-activating proteins (GAPs) catalyze GTP hydrolysis, returning Rabs to their inactive cytosolic state. Structurally Rabs contain a canonical GTP-binding domain that consists of five conserved guanine moiety binding motifs (G1-G5), Rab family specific motif (RabF), Rab subfamily special motif, geranylgeranylation motif, a hypervariable C-terminal domain, a C-terminal interacting motif, switch regions, and complementary determining regions (Pereira-Leal and Seabra, 2000; Pereira-Leal and Seabra, 2001; Pereira-Leal et al., 2003; Stein et al., 2012; Li et al., 2014). Rabs control the directed traffic of post-Golgi or endocytic vesicles modulating ciliary vesicle formation, centriolar uncapping, basal body maturation, cilia membrane extension, IFT and cargo trafficking to the cilium.

### 3.1 Rab8-Rab11

#### 3.1.1 Cilia assembly

Rab8 modulates the long-distance transport of *trans*-Golgi cargo (Wandinger-Ness and Zerial, 2014) and together with Rabin8, its GEF, and XM_037557 or TBC1D30, its GAP it modulates cilia biogenesis (Nachury et al., 2007; Yoshimura et al., 2007). Initiation of cilia assembly involves the transport particle protein complex II (TRAPPII) MTC and active Rab11^GTP^, a regulator of recycling endocytic vesicles to deliver Rabin8 to the preciliary vesicles (PCVs), which in turn recruits Rab8 (Knödler et al., 2010; Westlake et al., 2011). Interaction of TRAPPC14, a subunit of TRAPPII with Rabin8, Fbf1, and Cep83 promotes the engagement of the PCVs at the parent centriole (Cuenca et al., 2019). This is further augmented by Rab8 interaction with the distal centriolar proteins Cep164 (Schmidt et al., 2012), Ahi1 (Hsiao et al., 2009), and Talpid3 (Kobayashi et al., 2014). Fip3, a Rab11 effector binds Rabin8 to promote PCV trafficking, as well as stabilizes the Rab11-Rabin8 complex (Vetter et al., 2015; Walia et al., 2019). Recent imaging studies highlight the dynamic membrane conversion events in the Rab11-Rabin8-Rab8 cascade, wherein Rab11 and Rabin8 are depleted upon Rab8 loading (Saha et al., 2024). Moreover, Rab11 localization in the mature cilium was found to occur in a Rab8-dependent manner (Saha et al., 2024). Localization of Rab8 to the basal body is also facilitated by Chibby (Cby) which is recruited through its interaction with Cep164 (Burke et al., 2014) and Efa6a, a GEF for Arf6 GTPase (Partisani et al., 2021).

Ehd1, an Eps15 homology domain (Ehd) protein is a membrane-shaping factor that is recruited to PCVs and modulates their fusion into the ciliary vesicle (CV), which then encapsulates the distal surface of the parent centriole (Lu et al., 2015). This promotes localization of TZ proteins, Rpgrip1l, Tmem67 and Cep290 to the CV (Lu et al., 2015). Cep290 recruits Daz interacting zinc finger protein 1 (Dzip1), which in turn binds Rab8 and Cby to promote TZ assembly followed by CV extension (Zhang et al., 2015; Wu et al., 2020). Some studies suggest that Rab8 might be dispensable for the initial docking of ciliary vesicles, functioning only in subsequent stages of cilia membrane extension (Lu et al., 2015). Supporting this Rab8 was found to be non-essential for cilia formation in zebrafish and mammals (Sato et al., 2014; Aljiboury et al., 2023).

#### 3.1.2 Ciliary trafficking

**FIGURE 1:**
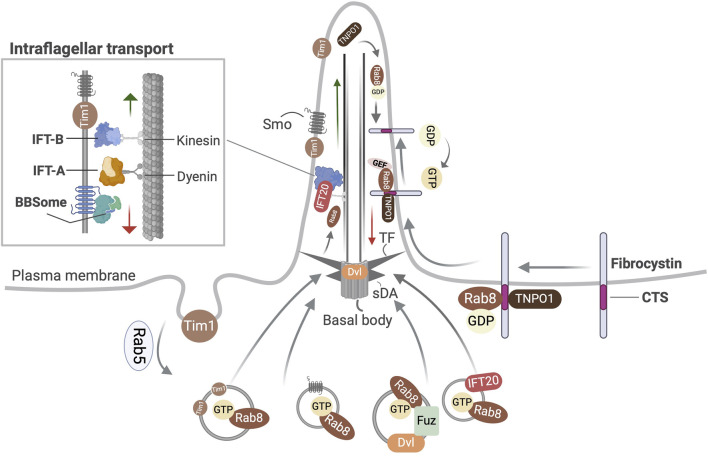
Overview of the transport functions of Rab8 related to primary cilia. Shown is Rab8 dependent transport of Smo, Dishevelled (Dvl), Tim1, and IFT20 into the primary cilium. In ciliated cells Fuzzy (Fuz) mediates the recruitment of Rab8 and Dvl to the primary cilium via the polarized trafficking route. Ciliary localization of fibrocystin involves the formation of a ternary complex with Rab8-TNPO1 at its ciliary targeting signal (CTS). TF, transition fibers; sDA, subdistal appendages.

Rab8 is implicated in the trafficking of polycystin-1 (PC1) (Ward et al., 2011), polycystin-2 (PC2) (Hoffmeister et al., 2011), the C-terminal fragment of fibrocystin (Follit et al., 2010), Smoothened (Smo), a transmembrane Hh receptor, Kim1, an apical membrane protein to the ciliary membrane (Figure 1). It assists in the transport of Dishevelled, a core planar cell polarity (PCP) component to the basal body (Zilber et al., 2013) and that of EB1, a cytosolic microtubule-binding protein into the cilium (Boehlke et al., 2010). The enrichment of Ift20 at the basal body also depends on Rab8 (Maharjan et al., 2020). The Golgi to cilia transport of rhodopsin, a GPCR occurs through directed rhodopsin transport carriers to the rod outer segment of vertebrate photoreceptors and involves the action of the Rab11–Fip3–Rabin8 dual effector complex and the interaction of Arf4 with Asap1, its GAP (Deretic et al., 1995; Mazelova et al., 2009; Bachmann-Gagescu et al., 2011; Wang et al., 2012; Vetter et al., 2015; Wang and Deretic, 2015). The transport of Kif17, a soluble kinesin-2 motor protein, Crumbs3 and retinitis pigmentosa 2 to the cilia membrane utilizes Importin-β2 and transportin 1 (TNPO1) that are conserved receptors in the nucleocytoplasmic trafficking machinery (Fan et al., 2007; Dishinger et al., 2010; Hurd et al., 2011; Kee et al., 2012) Based on studies with the known CTSs of fibrocystin, photoreceptor retinol dehydrogenase, rhodopsin and retinitis pigmentosa 2, a dynamic ternary complex of TNPO1-Rab8-CTS was deduced that can modulate the selective entry and retention of ciliary membrane proteins (Madugula and Lu, 2016). Once inside the cilium, GTP hydrolysis converts Rab8 to its inactive GDP-bound state leading to the release of its cargo. The Rab8^GDP^ is ubiquitinated and pre-emptively degraded by the protein quality control machinery (Takahashi et al., 2019) to prevent its accumulation which could exert harmful effects (Nachury et al., 2007; Yoshimura et al., 2007).

Abnormal *rab-8* expression in *C. elegans* compromises cilia structure and membrane transport (Kaplan et al., 2010). Dominant negative (T22N) form of *rab8* in *Xenopus laevis* rod photoreceptors results in retinal degeneration and accumulation of tubulovesicular structures at the cilium base in surviving outer rod segments (Moritz et al., 2001). Interestingly, *rab8* was dispensable while *rab11* and *rab35* were required for centrosome positioning and cilia formation during the development of the Kupffer’s vesicle (left-right organizer) in zebrafish (Aljiboury et al., 2023). In mammals, Rab8 occurs as Rab8a and Rab8b, which are isoforms encoded by different genes (Armstrong et al., 1996). *Rab8b* null mice are normal; *Rab8a* and *Rab8b* double knockout (KO) mice are also viable and largely normal except for the mis-localization of apical markers in selected tissues and do not show any ciliary abnormalities (Sato et al., 2014). This suggests that their ciliary functions may be redundant with other Rabs and likely explains the absence of human diseases associated with them. However, pathogenic variants in genes encoding Rab8 interacting proteins have been implicated in human disorders. Abnormal hexanucleotide repeat expansions in *C9orf72* that together with SMCR8 function as Rab8-GAP are implicated in approximately half the genetic cases of amyotrophic lateral sclerosis and frontotemporal dementia via the suppression of ciliogenesis and Hh signaling (Tang et al., 2024). Mutations in *OCRL1* that encodes for inositol-5-phosphatase, another Rab8 effector cause the X-linked oculocerebrorenal syndrome of Lowe (OCRL) or Lowe syndrome which manifests with abnormal primary cilia (Hou et al., 2011; Coon et al., 2012).

Mutations in *Leucine rich repeat kinase 2 (LRRK2*) that is a major regulator of both idiopathic (Nalls et al., 2014) and genetic forms of Parkinson’s Disease (PD) (Zimprich et al., 2004) cause hyperactivation of its kinase activity and are implicated in phosphorylation of Rab8a, Rab10 and Rab12 in cultured human and mouse cells, and mice (Ito et al., 2016; Steger et al., 2016; Thirstrup et al., 2017). Activating mutations in *LRRK2* cause aberrant accumulation of phosphorylated Rab8a at the centrosome leading to abnormal centrosomal cohesion and positioning, and defective cell polarity and migration (Madero-Pérez et al., 2018).

### 3.2 Rab10

**FIGURE 2:**
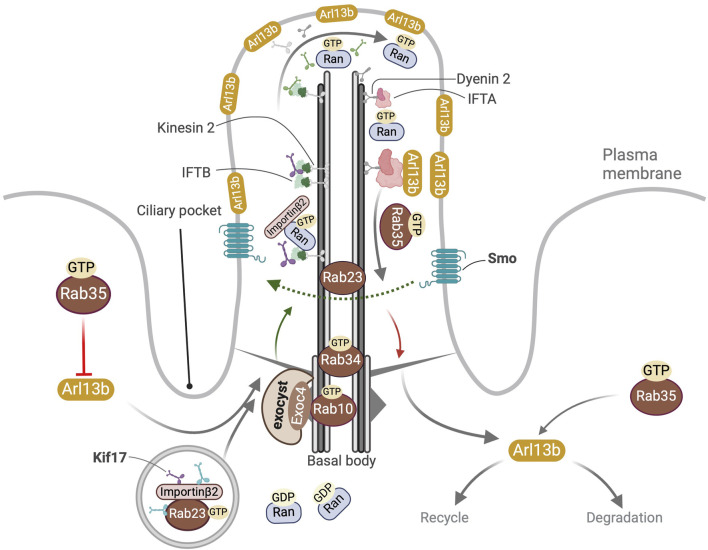
Overview of ciliary localization and functions of Rab10, Rab23, Rab34 and Rab35. Localization of GTP bound Rab10 and Rab34 at the basal body is shown. Active Rab10^GTP^ directly interacts with the exocyst component Exoc4. In the cytosol Rab23^GTP^ interacts with Kif17 and promotes its binding to importin-β2 to form a tripartite complex that then enters the cilia owing to the Ran^GTP^-Ran^GDP^ gradient between the primary cilium and the cytosol. In the cilium Ran^GTP^ binds importin-β2 leading to the release of Kif17 from Rab23 and the anterograde transport of Kif17 to the primary cilia tip. Rab23 also promotes recycling of ciliary Smo. Rab35^GTP^ controls the ciliary membrane composition by (a) inhibiting the import of Arl13b into the cilium (b) promoting the export of Arl13b out of the cilium (c) modulation of endocytic processes targeting Arl13b for degradation or recycling to non-ciliary destinations near the ciliary pocket.

Rab10 belongs to the same subfamily as Rab8 and is implicated in intracellular transport activities ranging from polarized exocytosis to endo-phagocytic processes (Chua and Tang, 2018). It localizes at the *trans*-Golgi, endoplasmic reticulum (ER), and at the base of primary cilia in renal epithelia. It colocalizes with Exoc3 and Exoc4, which are exocyst complex subunits, and directly interacts with Exoc4 at the basal body (Babbey et al., 2010) (Figure 2). Earlier studies suggest that Rab10 may be required for cilia biogenesis, as its depletion impaired ciliation more strongly compared to the combined inhibition of *Rab8a* and *Rab8b*, and the cumulative silencing of all three had the strongest effect (Sato et al., 2014). In contrast, RAB8A/B and RAB10 KO in hTERT-RPE cells had no effect on ciliogenesis (Oguchi et al., 2020). Another study suggested that Rab10 antagonizes primary ciliogenesis (Dhekne et al., 2018). Dennd2b, a Rab10 GEF that localizes at the basal body was found to suppress ciliogenesis by modulating Rab10-dependent recruitment of CP110, whereas it controlled cilia length by RhoA activation (Kumar et al., 2022).

Genetic disorders with Rab10 involvement include PD, wherein hyperactivated LRRK2 increases Rab10 phosphorylation, which in turn promotes its association with Rab interacting lysosomal protein-like 1 (RILPL1), a known suppressor of ciliogenesis (Dhekne et al., 2018) and accentuates the removal of ciliary membrane proteins (Schaub and Stearns, 2013). Disruption of *DENND2B* in a patient with subtelomeric *de novo* balanced translocation was associated with severe intellectual disability, muscular hypotonia, seizures, bilateral sensorineural hearing loss, submucous cleft palate, unilateral cystic kidney dysplasia, and other anomalies (Göhring et al., 2010).

### 3.3 Rab12

Rab12 is a key substrate of LRRK2 in the mouse brain that regulates the balance of ciliogenesis by controlling the centrosome/centriole homeostasis and inhibiting primary ciliogenesis. This depends on its interaction with LRRK2 and the phosphorylation of Rab10 (Dhekne et al., 2023). While *Rab12* KO mice are viable with no obvious developmental anomalies, examination of their brain slices revealed an increased frequency of ciliated cells and longer cilia in the striatum, even though the mean cilia volume remained unchanged; similar results were obtained from *Rab12* KO-derived primary astrocyte cultures *in vitro* (Li et al., 2024). This study showed that the overexpression of wild-type Rab12 led to centrosome amplification and aberrant cilia that resemble short “spikes” consisting of an enlarged lumen surrounding a defective axoneme. The overexpressed Rab12 promoted the colocalization of phosphorylated Rab10 and RILPL1 with endogenous LRRK2 at the amplified centrosomes (Dhekne et al., 2023). Similar results were obtained following Rab12 overexpression in RPE cells. Accordingly, when *LRRK2* was inhibited in astrocytes where a hyperactivating form of Rab12 was expressed, it abolished Rab10 phosphorylation and prevented the Rab12 overactivation-dependent deregulation of primary ciliation (Li et al., 2024). This study also showed that in primary cultures of astrocytes derived from mice carrying *LRRK2-G2019S*, a common PD mutation, the disruption of *Rab12* restored primary ciliation and centrosome homeostasis. Therefore, targeting the Rab12-LRRK2 complex could offer an attractive strategy for the amelioration of PD.

### 3.4 Other Rabs and primary cilia in the pathogenesis of PD

Other Rabs that are substrates of LRRK2 include Rab3, Rab35, and Rab43 (Steger et al., 2017). The pathology of PD involves the loss of dopaminergic neurons in the substantia nigra pars compacta region of the brain that projects into the dorsal striatum (Surmeier et al., 2014). Studies have shown that hyperactivating *LRRK2* mutations induce loss of cilia and Hh signaling in the cholinergic and parvalbumin interneurons and astrocytes in the mouse dorsal striatum, leading to the decreased expression of glia-derived neurotrophic factors, namely GDNF (Khan et al., 2021; Khan et al., 2024) and Neurturin (Lin et al., 2025) that are neuroprotective and essential for the survival of dopamine-synthesizing neurons.

### 3.5 Rab28

Rab28 carries a C-terminal CAAX motif instead of the common geranylgeranylation motif present in other Rab family members (Brauers et al., 1996) that gets prenylated and is required for its membrane retention at specific subcellular locations. It was identified as a ciliary protein in rat retina where it localized at the basal body and ciliary rootlet of photoreceptors (Roosing et al., 2013). In *C. elegans* its active GTP-bound form localizes at the periciliary membrane and requires the prenyl-binding protein Pde6d, a molecular chaperone, the BBSome and Arl-3 for import into the cilium and bidirectional IFT (Jensen et al., 2016; Akella et al., 2020). Ciliary Rab28 and the BBSome negatively regulate extracellular vesicles in a cilia-dependent manner in the sensory organs of *C. elegans* (Akella et al., 2020).

Biallelic *RAB28* null and hypomorphic alleles cause the rare cone-rod dystrophy (CRD; OMIM #615374) (Roosing et al., 2013; Riveiro-Álvarez et al., 2015). CRD is characterized primarily by the loss of cone photoreceptors or sometimes the loss of both cone and rod photoreceptors. It manifests with decreased visual acuity, color vision abnormalities, photo aversion, decreased sensitivity in the central visual field, progressive loss of peripheral vision, and night blindness (Hamel, 2007). *Rab28* KO mice exhibit progressive retina degeneration phenocopying the clinical features of CRD (Ying et al., 2018). These mice showed that Rab28 is required for shedding and phagocytosis of cone outer segment discs (Ying et al., 2018). In zebrafish *rab28* is required for both dawn and dusk outer segment phagocytosis peaks but not its basal levels (Carter et al., 2020; Moran et al., 2022).

A study with a homozygous missense single nucleotide variants (SNVs) in *RAB28* in a pair of siblings from a consanguineous family reported significant retinal degeneration and postaxial polydactyly (PAP) wherein the transcript levels of *Rab28* were unperturbed but its ciliary localization was abrogated (Jespersgaard et al., 2020). How Rab28 dysfunction causes PAP remains uncertain; however, this finding suggests that it may have a broader role in ciliopathies beyond CRD.

### 3.6 Rab34

Rab34 was first discovered in the Golgi apparatus (Wang and Hong, 2002). A CRISPR-based *in vitro* screen identified it as a positive regulator of primary ciliogenesis and Hh signaling (Pusapati et al., 2018). It was later identified as a ciliary protein using Ift27 proximity biotinylation in MEFs (Stuck et al., 2021). It modulates early cilia biogenesis by facilitating the fusion of PCVs into CVs (Xu et al., 2018). An extensive screen suggested that it was essential for serum starvation-dependent ciliogenesis in various cell lines e.g., hTERT-RPE1, NIH/3T3 cells, and MCF10A but unlike other Rabs its N-terminal and not the switch II region was required for ciliogenesis (Oguchi et al., 2020; Oguchi et al., 2022). Rab34 localizes at the basal body, and its GTP-bound form enters and resides in the cilium (Xu et al., 2018) (Figure 2). It localizes on the ciliary sheath in a specialized subdomain of assembling intracellular cilia and is pivotal for orchestrating initial steps in cilia membrane formation specifically in the intracellular but not the extracellular cilia biogenesis pathway (Ganga et al., 2021; Stuck et al., 2021).

*Rab34* ^*−/−*^ mice die perinatally, exhibiting polydactyly and craniofacial malformations (Dickinson et al., 2016; Xu et al., 2018). Rab34 plays both cilia dependent and independent roles in osteogenesis; its silencing in MC3T3-E1 murine preosteoblast cells strongly attenuated ciliation and Hh signaling that impaired cell proliferation, and osteoblast differentiation, in addition to the cilia-independent attenuation of type I collagen trafficking from the ER to Golgi apparatus (Yamaguchi et al., 2024). Recently, biallelic pathogenic nonsynonymous SNVs in *RAB34* have been linked with orofacial digital syndrome XX (OFDXX; OMIM #620718), wherein the patients manifest with features such as polydactyly/syndactyly, shortening of long bones, cleft lip and palate, micrognathia, cerebral, cardiac and anorectal anomalies (Bruel et al., 2023; Batkovskyte et al., 2024). Despite the *RAB34* pathogenic mutations being discerned as strong loss of function alleles that suppressed its expression and impaired primary ciliogenesis, the localization of the mutant RAB34 proteins at the basal body remained unperturbed (Bruel et al., 2023). Thus, a complete understanding of the molecular pathogenesis underlying OFDXX is still awaited.

### 3.7 Rab23

Rab23 was first identified in vertebrate embryogenesis for its function in dorsalization as an antagonist of Sonic hedgehog (Shh) that facilitates ventral cell fate specification (Chiang et al., 1996; Spörle et al., 1996). It localizes at the plasma membrane and endocytic vesicles, functioning downstream of Patched (Ptch) and Smo, which are Hh receptors but upstream of Gli transcription factors (Evans et al., 2003; Eggenschwiler et al., 2006). Rab23 activation requires Inturned and Fuzzy, which are GEFs and PCP pathway components functioning downstream of CV formation, and potentially parallel to Rab8 during primary cilia assembly (Gerondopoulos et al., 2019). Rab23 is required for cilia biogenesis (Yoshimura et al., 2007), where it controls the recycling of ciliary Smo (Boehlke et al., 2010) (Figure 2). Beyond the Shh pathway, it is also required for the ciliary trafficking of Kif17, a kinesin-2 motor protein by forming a complex with it and its nuclear transport adaptor Importin-β2 (Lim and Tang, 2015). It also promotes the IFT-B anterograde trafficking and Kif17-dependent targeting of D1-type dopaminergic receptors to the primary cilium (Leaf and Von Zastrow, 2015). It is also implicated in the ciliary targeting of other cargo, for example, subunits of olfactory cyclic nucleotide-gated channels (Jenkins et al., 2006). It indirectly controls the velocity of anterograde IFT as the latter depends on the number of active Kif17 motors and its dynamic equilibrium with Kif3ab motors when loaded on microtubules (Milic et al., 2017). Outside the cilium active Rab23 facilitates Kif17 migration to spindle poles; Rab23-Kif17-cargo complex modulates spindle organization via tubulin acetylation and drives actin cytoskeleton-mediated spindle migration during oocyte meiosis (Wang H. H. et al., 2019).

Pathogenic SNVs in *RAB23* are associated with Carpenter Syndrome (CS) a rare genetic disorder characterized by craniosynostosis of the face and skull, polydactyly, brachydactyly, heart and eye defects, structural and functional central nervous system (CNS) abnormalities (OMIM #201000) (Taravath and Tonsgard, 1993; Kadakia et al., 2014). Despite its well-established role in ciliogenesis, whether *RAB23* pathogenic variants cause abnormal ciliation in CS patients remains to be elucidated. Interestingly *Rab23* null mice (*open brain*) succumb mid-gestation due to Hh hyperactivation in the neural tube (Eggenschwiler et al., 2001).

### 3.8 Rab35

Rab35 is a conserved plasma membrane and endosomal protein with many essential cellular functions, for example, in the final steps of cytokinesis, endocytic cargo recycling, cytoskeleton modulation, and cell polarity (Kouranti et al., 2006; Klinkert et al., 2016). Its localization at the ciliary membrane in its active GTP-bound state is controlled by Dennd1b, its GEF, and Tbc1d10a, its GAP (Kuhns et al., 2019). This study also showed that Rab35 directly interacts with Arl13b, modulating ciliary membrane composition by augmenting the ciliary transport of Smo, PI(4,5)P_2_ and antagonizing the targeting of Arl13b and in turn that of INPP5E (Figure 2).

Rab35 was indispensable for ciliation in the left-right organizer in zebrafish, acting upstream of Rab11 for the apical membrane targeting of CFTR receptor in this context (Aljiboury et al., 2023). Recent studies identified MiniBAR, a dual Rab35 and Rac1 effector, which co-localizes with them at the vesicles destined for the primary cilia, controlling the ciliary targeting of proteins, such as Ift88 and Arl13b and regulating ciliogenesis through modulation of the actin cytoskeleton (Serres et al., 2023). This study also showed that MiniBAR depletion causes left-right asymmetry defects and ciliopathy-like features in zebrafish.

A recent study reported a single patient with a neurodevelopmental disorder harboring a missense mutation in *RAB35* which likely locks it in an inactive conformation, delaying cytokinesis, activating Arf6, and antagonizing primary ciliogenesis (Aguila et al., 2024). Consistent with its cellular roles, *Rab35* KO mice are embryonic lethal with cardiac edema; its conditional depletion in the kidney and ureter causes hydronephrosis and manifests with reduced primary cilia length, actin cytoskeleton disruption, abnormal cell polarization with loss of tight junctions, reduced adherens junctions, defective Arf6 epithelial polarity, cell death, and compromised differentiation (Clearman et al., 2024).

### 3.9 Rab19

In polarized MDCK cells, Rab19 localizes at vesicles accumulating at the apical cell surface and subsequently peripheral to the site of cortical actin clearing during ciliation (Jewett et al., 2021). This study showed that its depletion abrogated actin clearing above the centrosome blocking primary cilia assembly. Rab19-mediated orchestration of ciliary membrane growth occurred both in extracellular and intracellular pathways of ciliogenesis (Jewett et al., 2021). In its absence, cilia assembly stopped at the CV stage, even though the localization of Ift88, Ift140, and Rpgrip1l were unperturbed (Jewett et al., 2021). This study also showed that Rab19 interacts with Tbc1d4, a Rab-GAP as well as with every subunit of the homotypic fusion and vacuole protein sorting (HOPS) complex, a MTC that mediates vesicle and membrane fusion in late endosomes and lysosomes (van der Beek et al., 2019; Jewett et al., 2021). Subsequent work revealed that ablation of HOPS blocked basal body linked cortical actin clearing indirectly by retaining Rab19 on enlarged endosomes-lysosomes away from the basal body (Hoffman and Prekeris, 2023). Nevertheless, the direct involvement of HOPS and Rab19 interaction in primary cilia biogenesis remains inconclusive.

### 3.10 Rab5

Rab5 is a principal regulator of early endocytosis that is involved in early endosome biogenesis and fusion, multivesicular body formation, and endosomal trafficking (Gorvel et al., 1991; Bucci et al., 1992; Zeigerer et al., 2012). It was linked to primary cilia by studies in *C. elegans* that discerned it at the endocytic vesicles targeted to the periciliary membrane compartment at the ciliary base and a minor population of it was also noted in some axonemes (Kaplan et al., 2010; van der Vaart et al., 2015). It colocalized with Ocrl1 on internalized cilia-derived endosomes (Coon et al., 2012). Nevertheless, whether it is directly involved in primary ciliogenesis is uncertain as dominant negative *Rab5* attenuated the expression of Kim1 at the apical cell surface with no effect on the transport of ciliary proteins, such as Smo and EB1, and *Kim1* silencing also did not affect cilia biogenesis (Boehlke et al., 2010).

## 4 Rab-like GTPases

### 4.1 Rabl2

**FIGURE 3:**
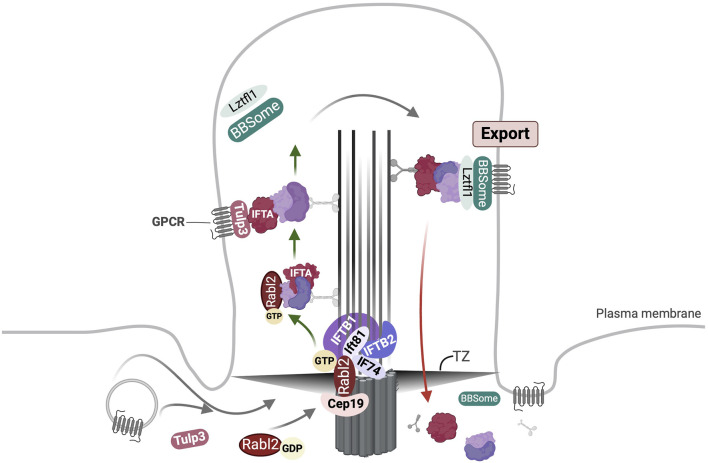
Overview of Rabl2 mediated ciliary transport. Rabl2^GTP^ is engaged at the basal body via interaction with Cep19 and Ift74/Ift81 heterodimer. The Cep19-Rabl2-Ift74/Ift81 complex facilitates the ciliary uptake of IFT particles lacking Ift25/Ift27. Following this majority of Rabl2 undergoes hydrolysis of bound GTP resulting in its replacement by Ift25/Ift27. However, some Rabl2^GTP^ may remain associated with the IFT-B trains and enter the cilium. Simultaneously, import of GPCRs is mediated by Tulp3 bound to IFT-A complex. Following anterograde transport the IFT trains are remodelled and motor proteins are exchanged at the ciliary tip (not shown). The export of GPCRs across the transition zone (TZ) is driven by BBSome bound to the IFT-B complex via Lztfl1 binding to Ift25/Ift27.

Rabl2 is an atypical Rab-like GTPase devoid of the C-terminal isoprenylation motif present in canonical Rabs (Homma et al., 2021). It is recruited to the basal body through interactions with Cep19 (Nishijima et al., 2017; Zhou et al., 2022), Cep164 and Cep83 (Dateyama et al., 2019) (Figure 3). It binds GTP by GEF-independent high-turnover nucleotide exchange cycle, and in this state interacts with the IFT-B1 core subcomplex (Taschner et al., 2016) via the Ift74/Ift81 heterodimer to recruit the IFT-B holo-complex from a pool of pre-docked IFT-B complexes, into the cilium (Kanie et al., 2017). Thus, the Cep19-Rabl2-IFT-B complex mediates the ciliary uptake of IFT moieties. The stable KO of *Cep19* or *Rabl2* decreased the frequency of IFT trains in motion but had no effect on IFT velocities (Kanie et al., 2017). The Ift74/Ift81 heterodimer serves as a Rabl2-specific GAP (Boegholm et al., 2023). Rabl2 has been suggested to promote the ciliary targeting of GPCRs such as Gpr161 and Htr6 (Dateyama et al., 2019). One model proposed that it indirectly regulates the BBSome-mediated export across the TZ by binding IFT-B particles (Duan et al., 2021). The authors in this study suggested that this is required for fine-tuning of signals; *Rabl2* ^*−/−*^ MEFs are unable to activate the Hh signaling in response to stimulation (Duan et al., 2021). Subsequent studies showed that Ift25/Ift27 and Rabl2 bind the Ift74/Ift81 heterodimer in a mutually exclusive manner (Zhou et al., 2022). In this work the authors suggested that Rabl2^GTP^-Cep19 at the basal body promotes the recruitment of IFT-B particles lacking Ift25/Ift27; following the hydrolysis of the bound GTP a majority of the Rabl2 is replaced by Ift25/Ift27, and any remaining Rabl2^GTP^ enters the cilium undergoing ciliary transportation while remaining associated with IFT-B machinery (Figure 3). Parallelly GPCRs are imported by Tulp3-IFT-A machinery (Zhou et al., 2022). Further they suggested that GPCRs are exported across the TZ by the BBSome which is connected to IFT-B particles via Lztfl1 binding it as well as the Ift25/Ift27 heterodimer (Figure 3) (Zhou et al., 2022).

*Rabl2* mutant *Chlamydomonas* lack flagella (Nishijima et al., 2017). Initially identified as an essential regulator of male fertility through a mutagenesis screen in mice, Rabl2 interacts with several IFT subunits, for example, Ift27, Ift81, and Ift172 for cargo delivery into the sperm tail (Lo et al., 2012). Consistent with this *Rabl2* mutant mice (*mot*) carrying the missense mutation (D73G) showed compromised sperm motility and were infertile (Yi Lo et al., 2016). The *Rabl2* ^*mot*^ mice exhibited ciliopathy features such as adult-onset obesity and fatty livers owing to defective glucose and lipid metabolism. Similar features, such as infertility, obesity, retinal degeneration, and polydactyly are also exhibited by *Rabl2* null mice despite overtly normal ciliation (Kanie et al., 2017; Duan et al., 2021).

Human patients with mutations in *RABL2* are not known likely due to the functional redundancy between RABL2A and RABL2B (Kanie et al., 2017). However, a deletion in *RABL2A* has been identified as a risk factor for infertility among Australian men (Jamsai et al., 2014). Analysis of rare and potentially deleterious SNVs in *RABL2* using mice has underscored its important role in cilia-mediated regulation of growth, left-right patterning, neural tube formation, limb development, and sperm motility (Ding et al., 2020). However, this study did not comment on the primary cilia architecture or frequency of ciliated cells in mutant *Rabl2* tissues though the sperm in the latter resembled that of *Rabl2* ^*mot*^ mice.

### 4.2 Rabl4/Ift27

Initially, Ift27 was discovered as part of the IFT-B core in *Chlamydomonas* by biochemical studies (Lucker et al., 2005). Later it was identified as an ortholog of Rab-like 4 (Rabl4). It retains four characteristic RabF motifs, which together with phylogenetic analysis suggested that it belonged to the ancestral Rab family in the LECA (Diekmann et al., 2011). It forms a nucleotide independent heterodimer with Ift25 (Bhogaraju et al., 2011) that localizes at the basal body and undergoes IFT in various organisms (Qin et al., 2007; Wang et al., 2009; Bhogaraju et al., 2011; Huet et al., 2014). Ift27 binding to Ift25 is required for the latter’s entry to the cilium but not its stability (Eguether et al., 2014). The Ift25/Ift27 heterodimer interacts with the Ift74/Ift81 heterodimer which tethers it to the IFT-B1 core subcomplex (Bhogaraju et al., 2011; Taschner et al., 2014). GTP binding to Ift27 is not required for the assembly of the Ift25/Ift27 heterodimer but promotes the engagement of the latter to IFT-B1 and its ciliary cycling (Eguether et al., 2014; Huet et al., 2014; Liu et al., 2023).

The requirement of Ift27 for cilia biogenesis is context dependent. In *Trypanosomes Ift27* silencing impedes cell growth and flagellum formation (Huet et al., 2014). In contrast, *Ift27* is dispensable for cilia assembly in mice as its ablation results in neonatal lethality without structural ciliary anomalies except in the sperm flagella (Zhang et al., 2017). Nevertheless, *Ift27* ^*−/−*^ mice display pleiotropic structural defects in the skeleton, heart, lung, and brain that are reminiscent of defective Hh signaling (Eguether et al., 2014). Studies in mice cells suggested that Ift27 independent of the IFT-B complex associates and stabilizes a nucleotide-free form of Arl6/Bbs3, followed by GTP loading and Arl6 activation that triggers the assembly of the BBSome-Arl6 coat on membrane surfaces (Jin et al., 2010), mediating the export of the BBSome (Liew et al., 2014). Ift25/Ift27 heterodimer associates with the BBSome via Lztfl1, a BBSome modulator and is required for the export of the BBSome, Lztfl1 and its cargoes, such as Ptch1, Smo and Gpr161 out of the cilium (Eguether et al., 2014). In *Chlamydomonas* as in mammals IFT27 is not required for IFT (Sun et al., 2021). Here LZTFL1 stabilizes IFT25/IFT27 that is essential for BBSome recycling at the cilia tips (Sun et al., 2021), independent of IFT27 nucleotide state and facilitates BBSome-dependent phospholipase D ciliary export (Liu et al., 2023).

While hypomorphic variants in *IFT27* cause Bardet-Biedl syndrome 19 (BBS19; OMIM #615996), a rare autosomal recessive condition characterized by ciliopathy characteristics, such as severe intellectual disability, polydactyly, renal failure, obesity, retinitis pigmentosa, and hypogonadism (Aldahmesh et al., 2014; Schaefer et al., 2019; Zhou et al., 2021); loss-of-function variants in *IFT27* have been associated with a lethal fetal ciliopathy with short ribs, hypodysplastic kidneys, imperforate anus and *situs invertus* with severely impaired ciliogenesis in patient-derived kidney sections (Quélin et al., 2018; Haïm et al., 2025).

### 4.3 Rabl5/Ift22

A ciliary role was first anticipated for Rab-like 5 (Rabl5) when *ifta-2,* its ortholog in *C. elegans* was found to localize in ciliated sensory neurons (Schafer et al., 2006). While *ifta-2* null worms did not exhibit typical ciliary defects they showed abnormal insulin-IGF-1-like signaling. Rabl5 is conserved across many ciliated organisms (Schafer et al., 2006; Silva et al., 2012). Studies showed that IFT22, a Rabl5 ortholog in *C. reinhardtii* associated with IFT-B complex, specifically with the IFT81/IFT74 heterodimer (Silva et al., 2012; Taschner et al., 2014). While in trypanosomes its recruitment to IFT-B1-1 subcomplex is essential for flallega formation; IFT and flagellation did not depend on its nucleotide state (Wachter et al., 2019). Work in *C. reinhardtii* suggests that active IFT22 interacts with GTP bound Arl6 to recruit the BBSome to the basal body (Xue et al., 2020). This study also revealed that ciliary uptake and cycling of the BBSome does not depend on IFT22. In contrast *Chlamydomonas* and *Trypanosoma brucei* (Adhiambo et al., 2009; Silva et al., 2012; Wachter et al., 2019) *IFT22* disruption did not affect ciliogenesis or ciliary signaling in hTERT-RPE cells (Takei et al., 2018).

## 5 Exocyst in membrane trafficking

The exocyst is a conserved ∼ 750 kDa heterooctameric complex that belongs to the Complexes Associated with Tethering Containing Helical Rods (CATCHR) class of MTCs that modulate tethering and fusion of post-Golgi and recycling endocytic vesicles at the plasma membrane (Ungermann and Kümmel, 2019). Its holo-complex comprises of eight subunits: Sec3, Sec5, Sec6, Sec8, Sec10, Sec15, Exo70 and Exo84 in budding yeast that are termed EXOC1-8 in mammals (Hsu et al., 1996; TerBush et al., 1996). Each exocyst subunit contains the characteristic CATCHR domain and CorEx (core of exocyst) motif, an N-terminal coiled-coil followed by a rod composed of short antiparallel helical bundles (Mei et al., 2018). The mammalian exocyst is an octamer that is dynamically assembled from two subcomplexes (SC), SC1 (EXOC1-4) and SC2 (EXOC5-8) that are pre-assembled at the vesicles and likely arrive together at the plasma membrane (Ahmed et al., 2018; Maib and Murray, 2022).

Exocyst recruitment to secretory vesicles involves Sec15/Exoc6 binding Sec4p/Rab33b (Guo et al., 1999) and Sec2 its GEF (Medkova et al., 2006). Myo2, the yeast myosin V binds both Exoc6 and Rab33b (Jin et al., 2011). For vesicle delivery to polarized membranes, the conversion of PI4P to PI(4,5)P_2_ by Arf6 triggers exocyst recruitment and both SC1 and SC2 independently bind to the membrane PI(4,5)P_2_ (Maib and Murray, 2022). Studies in yeast showed that Sec3/Exoc1 (Zhang et al., 2008) and Exo70/Exoc7 (He et al., 2007) bind PI(4,5)P_2_ in the target membranes to engage the exocyst at the site of exocytosis. Recent studies also showed that the exocyst not only mediates SNARE assembly (Sivaram et al., 2005; Dubuke et al., 2015; Yue et al., 2017) but functions downstream driving complete membrane fusion and mixing of vesicle contents (Lee et al., 2024).

### 5.1 Exocyst and primary ciliogenesis

The exocyst is required to support numerous cellular functions, such as cell polarity (Polgar et al., 2015), migration (Zuo et al., 2006), autophagy (Bodemann et al., 2011), and cytokinesis (Gromley et al., 2005). It was first shown to localize to the primary cilium in MDCK cells (Rogers et al., 2004) and the ciliary stalk of photoreceptors in the frog retina (Mazelova et al., 2009). Using live imaging Rivera-Molina *et. al.,* showed that in the presence of serum, the exocyst was enriched at the ciliary pocket of internal cilia; upon serum withdrawal, as the cilia are recycled to the cell surface, the exocyst was redistributed at its base (Rivera-Molina et al., 2021). In hTERT-RPE1 cells, SEPTIN9 a filamentous GTPase was shown to activate RhoA GTPase that in turn recruited the exocyst to the ciliary base and the depletion of either SEPTIN9 or the exocyst abolished the TZ and cilia assembly (Safavian et al., 2023).

**FIGURE 4:**
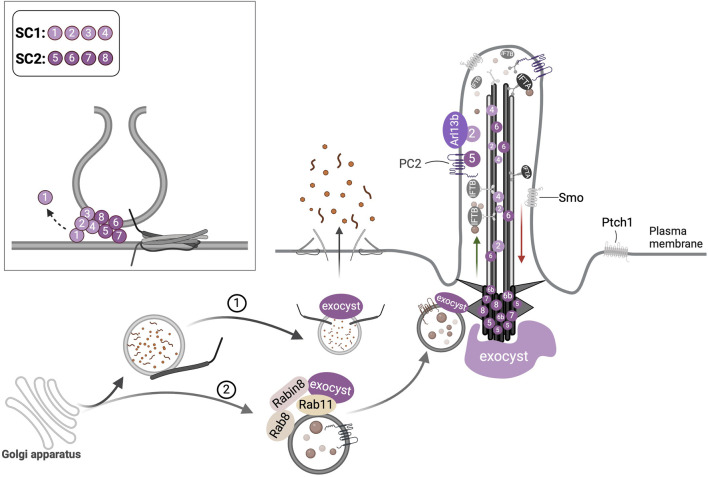
Ciliary transport functions of the exocyst complex. (1) Post-golgi vesicles in the secretory pathway are tethered at the plasma membrane by the exocyst that mediates exocytosis. (2) Vesicles with ciliary cargo are targeted to the cilium by the interaction of Exoc6, an exocyst subunit (not shown here) with Rab11 and Rabin8. The exocyst localizes at the primary cilium and periciliary regions; its subunits Exoc2, Exoc4, and Exoc6 have been detected at the axoneme, and others such as Exoc5, Exoc6b, Exoc7, and Exoc8 have been noted at the basal body. The direct binding of Arl13b to Exoc2 and PC2 to Exoc5 is illustrated. The inset depicts the formation of the heterooctameric exocyst complex at the target membranes by the dynamic assembly of its subcomplexes (SC), SC-1 and SC-2 which mediates tethering of the vesicles, SNARE assembly followed by membrane fusion.

While some exocyst subunits Exoc2, Exoc4 (Fogelgren et al., 2011; Seixas et al., 2016) and Exoc6 (Feng et al., 2012) localize to the axoneme, others such as Exoc3 (Yeaman et al., 2001), Exoc5 (Seixas et al., 2016), Exoc6b (Guleria et al., 2024), Exoc7 and Exoc8 (Seixas et al., 2016) occur at the basal body and periciliary regions (Figure 4). Some exocyst components also interact with ciliary proteins: Exoc5 binds Arl13b, Ift20, Ift88, and PC2 (Fogelgren et al., 2011); Exoc2 and Exoc3 directly interact with Arl13b and genetic interaction was reported between Arl13b and Exoc5 (Seixas et al., 2016). Ift20 interacts with Exoc4 and Exoc7 (Monis et al., 2017). The exocyst was also shown to cooperate with primary cilia in extracellular vesicle production in MDCK cells (Zuo et al., 2019a).

Studies *in vitro* and animal models underscore the essentiality of the exocyst for ciliogenesis (Table 2). Many studies targeted *Exoc5* as it is indispensable for exocyst assembly. In zebrafish, *exoc5* morphants phenocopied *pc2* depletion, and the manipulation of *Exoc5* in MDCK cells led to aberrant ciliary assembly and mechanosensation defects reminiscent of Autosomal dominant polycystic kidney disease (ADPKD) (Fogelgren et al., 2011). An *exoc5* loss of function mutant in zebrafish showed gross abnormalities of the brain, retina, and heart consistent with ciliopathies (Lobo et al., 2017). The complete ablation of *exoc5* in zebrafish resulted in severe cardiac dysfunction and succumbed 4 days post fertilization; *Exoc5* endocardial cell-specific silencing in mice showed dysmorphic aortic valves abrogating ciliogenesis and allied ciliary signaling (Fulmer et al., 2019). In MDCK cells the knockdown of *Exoc5* or mutating its CTS suppressed ciliogenesis and its overexpression led to elevated ciliation (Zuo et al., 2019b). Other studies also recapitulated abnormal ciliation following *Exoc5* knockdown in MDCK cells, though lumen formation was unaffected in 3-dimensional cultures; mice with kidney-specific knockdown of *Exoc5* exhibited PCP defects with suppressed primary ciliation (Polgar et al., 2015). Seixas et al. showed that *Exoc5* conditional inhibition in mouse kidneys caused high mortality and the surviving animals had a range of abnormalities including enlarged kidneys with cysts, significantly fewer and shorter cilia and overall reduced Arl13b protein levels (Seixas et al., 2016). The Exoc5 CTS was required for proper exocyst function in ciliation and its mutation led to defective cystogenesis and tubulogenesis in MDCK cells (Zuo et al., 2019b).

**TABLE 2:** Exocyst and cilia in organismal development.

Gene	Mutant type	Organism/Cell type	Cilia	Phenotype	Reference
*Exoc1*	*Exoc1* ^*−/−*^	Mouse	NA	Peri-implantation lethal	Mizuno et al. (2015)
	*Nanos-Cre* ^*+*^ *;Exoc1* ^*fl/fl*^	Mouse	NA	Impaired spermatogenesis	Osawa et al. (2021)
*Exoc3*	*PF4-Cre+;Exoc3* ^*−/−*^	Mouse	NA	Defective platelet aggregation, integrin activation, increased arterial thrombosis	Walsh et al. (2021)
*Exoc4*	*Exoc4* ^*−/−*^	Mouse	NA	Embryonic lethal – survive to primitive streak stage	Friedrich et al. (1997)
*Exoc5*	*exoc5* KD	Zebrafish	Unaffected	Small body size and eyes, edema, curly tail up, and defective left-right patterning	Fogelgren et al. (2011)
	*Exoc5* misexpression	MDCK	Loss of cilia with *Exoc5* KD and longer cilia with *Exoc5* OE	Altered mechanosensation, increased cell proliferation in *Exoc5* KD	
	*Exoc5* shRNA KD	MDCK	Lower frequency, shorter and stubby	Abnormal cyst formation with increased apoptosis on the outside of the cyst and increased sensitivity to apoptotic cues, PCP defects	Polgar et al. (2015)
	*Ksp-Cre* ^*+*^ *; Exoc5* ^*fl/fl*^	Mouse		neonatal lethality, ∼1% of mice survived to 3 weeks, cystic kidney with dilated kidney tubules, renal tubules showed fragmentation and shrinkage due to apoptosis	
	*exoc5* KD	Zebrafish	NA	Phenotypes related to abnormal ciliation	Seixas et al. (2016)
	*Ksp1.3-Cre* ^*+*^ *; Exoc5* ^*fl/fl*^	Mouse	Suppressed Arl13b protein levels	Uterobilateral ureter obstruction leading to early death, 10-week-old pups with smaller fibrotic kidneys, numerous cysts	
	*Exoc5* misexpression	MDCK	*Exoc5* KD: absent *Exoc5* OE: longer	Reduced extracellular vesicle production with *Exoc5* KD	Zuo et al. (2019a)
	*Exoc5*-CTS mutant	MDCK	Fewer, length unaffected	Inhibited cystogenesis and tubulogenesis	Zuo et al. (2019b)
	*exoc5* KO	Zebrafish	Absent	Hydrocephalus, smaller eyes, pericardial edema	Lobo et al. (2017)
	*Rho-Cre+; Exoc5 fl/fl*	Mouse		Disorganized photoreceptor outer segments, shorter photoreceptor outer segment length	
	*exoc5* ^*−/−*^	Zebrafish	NA	Pericardial edema, severe cardiac outflow obstruction, death at 4 dpf	Fulmer et al. (2019)
	*NfatC1-Cre* ^*+*^ *; Exoc5f/f*	Mouse	Reduced length and frequency	Ventricular septal defects, apical coronary vascular haemorraging, bicuspid aortic valve, valvular stenosis, and valvular calcification	
*Exoc6*	*Exoc6* misexpression	hTERT-RPE	Shorter	*Exoc6* OE cells arrested recycling endosomal trafficking	Feng et al. (2012)
	*Exoc6* ^*−/−*^ *(hbd)*	Mouse	NA	Inhibition of erythroid iron assimilation	Lim et al. (2005), White et al. (2005)
*Exoc6b*	*Exoc6b KD*	ATDC5	Reduced frequency of ciliated cells and shorter cilia	Impaired chondrocyte differentiation	Guleria et al. (2024)
*Exoc7*	*Exoc7* ^*−/−*^	HeLa and adipocytes	NA	Abrogation of insulin-stimulated GLUT4 translocation	Wang et al. (2019b)
	*exoc7* null	*C. elegans*	NA	Slow growth, Uncoordinated movement Impaired response to chemical, mechanical, and thermal stimuli	Jiu et al. (2012)
*Exoc8*	*exoc8* null	*C. elegans*	NA	Same as *exoc7* null worms	Jiu et al. (2012)

KD: knockdown; OE: overexpression; NA: not available.

Studies in MDCK cells, zebrafish, and mice also revealed that Cdc42, a small GTPase that interacts with the exocyst colocalizes with Exoc5 at the primary cilium and acts in concert with it for polarized secretion and primary ciliogenesis (Zhang et al., 2001; Zuo et al., 2011). Accordingly, *exoc5* morphant zebrafish have small eyes (Fogelgren et al., 2011) and show loss of photoreceptor cilia, similar to that observed following *cdc42* silencing (Choi et al., 2013). *Cdc42* depletion in the mouse kidney led to polycystic kidney disease, aberrant cystogenesis with fewer and abnormal cilia surrounding the cysts (Choi et al., 2013). The knockdown of *Tuba*, a Cdc42-specific GEF suppressed primary ciliation in MDCK cells and *tuba* morphant zebrafish exhibited severe renal abnormalities and defective cilia (Baek et al., 2016). *Exoc6* silencing in hTERT-RPE1 cells produced shorter cilia (Feng et al., 2012). Inhibition of *Exoc6b,* a paralog of *Exoc6* in ATDC5 pre-chondrocytes strongly suppressed both the frequency of ciliated cells, cilia length, and deregulated chondrocyte differentiation (Guleria et al., 2024). In *C. elegans exoc7* and *exoc8* loss of function mutants showed behavioral defects akin to that observed with dysfunctional ciliogenesis albeit without any structural ciliary defects (Jiu et al., 2012).

### 5.2 Exocyst in ciliary trafficking

The delivery of fibrocystin and PC2 to the ciliary membrane depends on the exocyst (Monis et al., 2017). Similarly, rhodopsin targeting to outer photoreceptor segments requires exocyst function. Accordingly, retina-specific *Exoc5* KO in mice led to the loss of photoreceptor outer segments and their associated cilia, as well as impaired vision (Lobo et al., 2017). Exoc6 directly binds Rabin8 in hTERT-RPE1 cells indicating the involvement of the exocyst in vesicle targeting during cilia assembly (Feng et al., 2012). In mammalian cells, the trafficking of soluble ciliary proteins, such as Gli2/3 and Lkb1 to the primary cilium was dependent on the exocyst and involved Rab14, Rab18, Rab23, and Arf4 (Niedziółka et al., 2024).

### 5.3 Exocyst and ciliopathies

Rare pathogenic mutations in several exocyst subunits are linked to monogenic diseases with abnormal cilia. *EXOC2* biallelic mutations cause a neurodevelopmental disorder with dysmorphic facies and cerebellar hypoplasia (NEDFACH; OMIM #619306), wherein patient-derived fibroblasts EXOC2 expression was strongly suppressed leading to impaired vesicle fusion and exocytosis, and defective ARL13B localization in the cilium (Van Bergen et al., 2020). Homozygous null mutations in *EXOC6B* are linked to spondyloepimetaphyseal dysplasia with joint laxity type 3 (SEMD-JL3; OMIM #618395) that manifest largely with skeletal anomalies, such as hyperlaxity of joints and multiple joint dislocation, although some SEMD-JL3 patients do exhibit structural brain abnormalities and cognitive deficits (Girisha et al., 2016; Campos-Xavier et al., 2018; Simsek-Kiper et al., 2022). Our studies showed that *EXOC6B* mutant patient-derived fibroblast cells were defective in primary ciliogenesis (Simsek-Kiper et al., 2022). Similarly silencing *Exoc6b* in ATDC5 cells inhibited cilia assembly and led to abnormal chondrocyte differentiation (Guleria et al., 2024). Partial loss of function mutations in *EXOC7* cause neurodevelopmental disorder with seizures and brain atrophy (NEDSEBA; OMIM #619072). Truncating mutations in *EXOC8* are linked to neurodevelopmental disorder with microcephaly, seizures, and brain atrophy (NEDMISB; OMIM #619076). *Exoc7* null mice are embryonic lethal (Coulter et al., 2020). The *exoc7* loss of function zebrafish manifest ciliopathy features, such as edema and small eyes, as well as showed microcephaly owing to elevated apoptosis that led to a reduction in the number of neural progenitors in the telencephalon of mutant zebrafish (Coulter et al., 2020).

## 6 Future perspectives

Here we reviewed the involvement of Rabs, Rab-like factors, and the exocyst in ciliary trafficking and biogenesis. Based on the existing findings we note the below:

The Rab8-Rabin8-Rab11 cascade is implicated in early ciliogenesis. While Rab8 has been extensively studied we know comparatively little about the essentiality of Rab11 in this process. Engineering mutant Rab11 locked in GTP and GDP bound conformations by gene editing *in vitro* and animal models may help to shed light on this.

Activating mutations in *LRRK2* linked to PD have been shown to involve several Rab substrates that function at least in part at the cilium, including Rab8, Rab10, and Rab12. More studies are required to uncover not only all the Rabs involved but also to elucidate their precise roles. Furthermore, it is unclear why *LRRK2*-dependent cilia loss is cell-type specific, affecting astrocyte and cholinergic interneuron cilia but not those in medium spiny neurons of the dorsal striatum (Khan et al., 2024; Lin et al., 2025). It would be interesting to examine this problem from the lens of the types of ciliary proteins differentially trafficked in cells sensitive to versus those that are not to *LRRK2* over activating mutations.

For many years *RAB23* mutations have been linked to CS which manifests with ciliopathy-like features. However, ciliation and trafficking abnormalities in them remain to be elucidated. The tissue-specific analysis of cilia in major organ systems affected in CS, for example, the CNS, skeleton, and heart may help to shed light on ciliary defects and pathologically relevant RAB23 cargo. In terms of pathomechanisms, future work could focus on the genotype-phenotype correlation of *RAB28* mutations linked with CRD and PAP.

Secretomics in HeLa cells has shown that the exocyst is required for constitutive secretion (Pereira et al., 2023). It would be interesting for future studies to examine if exocyst-dependent ciliary trafficking is required in this process.

Conditional depletion of *Exoc5* in podocytes, a unique cell type in the kidney glomerulus damages its structure leading to proteinuria and renal failure, without primary cilia involvement (Nihalani et al., 2019). In this study, the authors argue that podocyte-specific *Ift88* silencing also does not lead to renal abnormalities. This is contrary to another study that reported a homozygous missense mutation in *IFT139* that encodes for TTC21B, an IFT-A retrograde complex subunit in a patient with Focal segmental glomerulosclerosis wherein *IFT139* silencing was suggested to result in abnormal primary cilia with aberrant aggregation of IFT-B components at the tips (Huynh Cong et al., 2014). Further studies will be needed to resolve the physiological relevance of the exocyst and cilia axis in podocytes.

The contribution of cilia is unclear in several exocyst-linked genetic diseases, for example, *EXOC7* and *EXOC8* linked NEDSEBA and NEDMISB, respectively. More detailed analysis is warranted to examine the molecular pathomechanisms in these disorders, including the disruption of primary ciliogenesis in them.

It is likely that not only the exocyst but other MTCs such as HOPs also interplay with distinct Rabs to modulate primary ciliogenesis. Future studies should examine these interactions to unveil their unique requirements in ciliary trafficking.

Finally, improving our understanding of the crosstalk and combinatorial action between different constituents of the membrane trafficking machinery will deepen our understanding of the sophisticated biological mechanisms underlying cilia biogenesis and function, as well as their clinical implications.
